# A Review of Circulating Tumor DNA as a Biomarker Guide for Total Neoadjuvant Therapy in Patients with Locally Advanced Rectal Cancer

**DOI:** 10.1007/s12029-022-00906-z

**Published:** 2023-01-31

**Authors:** Jehan Yahya, Miriam Baber, Nima Nabavizadeh, Shaun M. Goodyear, Adel Kardosh

**Affiliations:** 1 Department of Radiation Medicine, Oregon Health & Science University (OHSU), Portland, OR USA; 2 University of Kansas School of Medicine-Wichita, Wichita, KS USA; 3 Knight Cancer Institute, OHSU, Portland, OR USA; 4 Division of Hematology and Oncology, School of Medicine, OHSU, Portland, OR USA

**Keywords:** Biomarkers, Cell free, Circulating tumor, Total neoadjuvant, Locally advanced rectal cancer

## Abstract

**Purpose:**

Non-operative management of patients with locally advanced rectal cancer (LARC) is emerging as a popular approach for patients that have no evidence of disease following neoadjuvant therapy. However, high rates of local recurrence or distant metastases have highlighted the urgent need for robust biomarker strategies to aid clinical management of these patients.

**Methods:**

This review summarizes recent advances in the utility of cell-free (cf) and circulating tumor (ct) DNA as potential biomarkers to help guide individualized non-operative management strategies for LARC patients receiving neoadjuvant therapy.

**Results:**

Liquid biopsies and the detection of cfDNA/ctDNA is an emerging technology with the potential to provide a non-invasive approach to monitor disease response and improve the identification of patients with LARC that would best benefit from non-operative management.

**Conclusions:**

Substantial work is still needed before cfDNA/ctDNA monitoring can be widely adopted in the clinical setting. Studies reviewed herein highlight several areas of opportunity for improving the effectiveness and utility of cfDNA/ctDNA for managing patients with LARC.

## Introduction

Total mesorectal excision (TME) remains central to the management of patients with locally advanced rectal cancer (LARC) and is often preceded by neoadjuvant treatment (NAT) that includes radiation therapy (RT) with or without sensitizing chemotherapy[1, 2]. This strategy has evolved to include systemic chemotherapy administered before or after chemoradiation (CRT). Termed total neoadjuvant therapy (TNT), the approach is shown to achieve excellent local tumor control and long-term survival [3–6]. While TNT increases treatment compliance and can reduce time to ileostomy closure, TME is associated with significant perioperative morbidity and mortality, and sexual, urinary, and bowel dysfunction, which can dramatically reduce a patient’s quality of life (QOL) [7]. Mounting evidence shows that 20–30% of patients with LARC undergoing TNT achieve a clinical complete response (cCR) that can be non-operatively managed and have lower local recurrence and improved survival rates compared to those that did not attain a cCR [8–11]. Organ preservation with deferral of upfront TME for patients with cCR to TNT has subsequently emerged as an exciting clinical strategy [8–14]. However, among patients with cCR that are non-operatively managed, approximately 16–34% experience localized disease recurrence, and 8–10% develop metastases within the first 3 years [8, 15, 16]. This highlights several practical challenges to managing non-operative patients; notably, the lack of robust tests to predict which cCR patients would most benefit from non-operative management. Presently, frequent follow-up monitoring visits involve several different imaging modalities (e.g., MRI, PET/CT), digital-rectal exams, and endoscopies, as well as assessment of carcinoembryonic antigen (CEA) and/or CA-19–9 in blood, but these are altogether not reliably predictive [17–21].

Improved strategies for selecting the patients with LARC who may most benefit from non-operative management are urgently needed. The presence of cell-free DNA (cfDNA) circulating in the blood has long been known, but its implication in cancer was not realized until advanced sequencing techniques such as quantitative polymerase chain reaction (qPCR) and next-generation sequencing (NGS), among others, became widely available [22–24]. cfDNA is shed by both non-malignant (predominantly hematopoietic cells) and malignant cells, within which is the smaller fraction of tumor-derived DNA (i.e., circulating tumor DNA [ctDNA]). Isolation of cfDNA from the blood plasma remains the most preferred source, but is also present in saliva, urine, pleural fluid, and cerebrospinal fluid among other body fluids [25–28]. The concentration of cfDNA/ctDNA varies by tumor type, disease staging, tumor burden, and vascularization, as well as treatment type and response [23, 28–33]. The primary types of information that can be gathered from ctDNA analysis are evaluating the genomic sequences in tumors and quantifying tumor burden [34]. Studying the genomic sequences of tumors may allow for more targeted therapy options, while quantification of tumor burden can have prognostic and early detection implications. Furthermore, the methylation (or silencing) of genes studied during cfDNA/ctDNA analysis may provide prognostic value [35]. This has catapulted efforts to exploit cfDNA/ctDNA as a non-invasive approach to evaluating a patient’s response to cancer therapy. Considerable efforts have examined colon cancer, but the role of cfDNA/ctDNA in LARC remains understudied. This review summarizes prominent studies examining cfDNA and ctDNA as potential biomarkers to help guide individualized non-operative management strategies for LARC patients receiving NAT.

## cfDNA Biomarkers to Guide TNT for Rectal Cancer

Early reports using PCR showed the feasibility of cfDNA detection in rectal cancer and its correlation to clinical outcomes. Using qPCR to quantify 18S gene plasma levels, Zitt et al. [36] showed that among patients with LARC that received neoadjuvant CRT (45 Gy, 5-fluorouracil [5FU]), postoperative levels of cfDNA were significantly lower in LARC patients achieving ypT0-T2 staging compared to those with ypT3-T4 staging. In an effort to better discriminate non-tumor and tumor cfDNA origins, Angostini et al. used a cfDNA integrity index based on a ratio of short (115 bp, non-tumoral) and long (247 bp, tumoral) fragments of Alu repeats [37]. In plasma samples collected post-CRT, cfDNA integrity index was significantly lower among LARC patients with histopathological response after surgery (i.e., tumor regression grades [TRG] 1–2) than non-responders (i.e., TRG 3–5) [37]. Likewise, the integrity index of cfDNA derived from the targeted amplification of 400 bp and 100 bp fragments of the β-actin gene was also similarly decreased (from baseline) in LARC patients after CRT, and correlated with response [38].

Attempts to provide a more rapid and cost-effective quantitation of cfDNA levels have sought to employ a direct fluorescent assay (DFA) using SYBR Gold dye [39–41]. Truelsen et al. examined cfDNA levels using direct fluorescent assay (DFA) across 76 patients with LARC receiving neoadjuvant short-course RT (25 Gy/5 fractions/5 weeks), or long-course RT (50.4 Gy/28 fractions/5 weeks) with or without capecitabine (850 mg/m^2^ BID) [39]. Blood samples collected at baseline had the highest levels of cfDNA that significantly decreased during the course of treatment. Decreases in cfDNA levels (from baseline) greater than the 75th percentile were significantly associated with patients achieving pCR, and corresponded with improved RFS (HR 3.23, 95% CI 1.38–7.56, *p* < 0.05) but not OS (HR 1.75 (95% CI 0.48–6.38) [39]. In a similar study, Schou et al. showed that DFA-based quantification of plasma cfDNA levels ≥ 75th percentile was associated with LARC patients having a significantly higher risk of RFA (HR 2.43, 95% CI 1.27–4.7, *p* = 0.015) when compared to those with cfDNA < 75th percentile. cfDNA levels ≥ 75th percentile also portended a greater risk for local or distant recurrence, and shorter time to recurrence (HR 2.48, 95% CI 1.3–4.8, *p* = 0.007) [40].

Altogether, cfDNA quantification is a promising prognostic tool for improving the management of patients with LARC; however, the broad examination of nucleic acid content in the blood is not tumor specific, and underlying patient comorbidities as well as DNA contamination from lymphocytes can confound interpretation of findings [42–44]. The clinical utility of profiling cfDNA levels alone is likely insufficient, but can improve detection of mutations, as well as inform alternative non-genetic cancer monitoring strategies (e.g., methylome, fragmentomics) [45–47]. This is particularly important in LARC, where lower tumor fractions following the completion of neoadjuvant therapy can limit ctDNA quantification [48, 49].

## ctDNA as a Biomarker to Guide TNT for Rectal Cancer

Studies profiling ctDNA have largely focused on the detection of tumor-specific mutations using PCR-based (e.g., droplet digital [ddPCR], BEAMing PCR) and/or NGS-based technologies to evaluate either pre-defined target mutations or patient-specific mutations, which may or may not be informed by the patient’s tumor tissue. The limitations of these technologies have been extensively reviewed [50–52]. Though not patient-specific, pre-defined targeted panels have the benefit of amplifying ctDNA for common tumorigenic mutations, whereas patient-specific monitoring has the advantage of providing a personalized technique for in-depth assessment of mutations present in an individual patient’s tumor [52, 53]. The utility of ctDNA at different stages of the neoadjuvant treatment management of LARC patients is summarized below.

Pazdirek et al. used a PCR-based panel of six mutations (KRAS, BRAF, PIK3CA, CTNNB1, APC, and TP53) to detect ctDNA in a cohort of 36 patients with LARC undergoing neoadjuvant CRT and surgery [54]. Somatic mutations were identified in the tumors of 33 of 36 patients, but the presence of ctDNA prior to CRT was only observed in 7 of these 33 patients (21.2%). A comparison of ctDNA status (i.e., positive vs. negative) showed that ctDNA positivity prior to CRT was significantly associated with a worsened RFS and OS [54]. Even so, the concern remains that the six mutation signature may limit the detectability of ctDNA at baseline.

Ji et al. examined the prognostic value of tumor mutational burden in the blood (bTMB) of LARC patients undergoing neoadjuvant CRT and surgery [55]. Correlation between bTMB level and tumor recurrence among 46 LARC patients showed that those with low bTMB levels (i.e., TMB ≤ 10 Mb) at baseline were associated with an increased risk of recurrence, whereas patients with high bTMB levels (TMB > 10 Mb) after surgery had a greater risk of recurrence. The prognostic value for when bTMB ≤ 10 Mb yielded a sensitivity and specificity of 100% and 70.3%, respectively. Interestingly, measures of serum cytokine levels showed significantly higher levels of interleukin (IL)-1β, IL-2, interferon (IFN)-γ, IFNα2, and macrophage inflammatory protein 1β (MIP-1β) in baseline samples from patients with bTMB > 10 Mb compared to those in the low bTMB group. Post-operatively, patients with bTMB < 10 Mb had higher levels of granulocyte–macrophage colony-stimulating factor (GM-CSF), IL-9, and tumor necrosis factor β (TNF-β), which was associated with improved RFS [55]. These findings suggest a broader role for assessing immune activity as part of cfDNA monitoring.

Zhou et al. reported on the ctDNA characteristics of 104 patients with LARC that underwent neoadjuvant CRT followed by surgery [48]. A targeted NGS panel was used to examine ctDNA from blood samples at baseline, during CRT, pre-surgery (~ 7 weeks after CRT), and post-surgery (~ 1 month post-surgery). Among the 104 patients, a total of 1098 mutations were identified in tumor tissues. At baseline, ctDNA was detected in 75% of cases, but dramatically decreased during the course of treatment, with only 10.5%, and 6.7% of cases having detectable ctDNA pre- and post-surgery, respectively. While neither ctDNA positivity at baseline or during CRT correlated with response, it was noted that none of the 29 patients that achieved pCR had detectable pre-surgery ctDNA. This rate of pre-surgery ctDNA positivity was significantly lower in those with TRG 0–1 than TRG 2–3 (*p* < 0.001). Importantly, ctDNA positivity across all four timepoints was associated with a shorter metastasis-free survival, notable of which was that the median variant allele frequency (VAF) of baseline ctDNA > 1% was a significant predictor of early distant metastasis prior to the starting treatment (HR, 6.549; *p* = 0.001). Interestingly, though elevated baseline CEA or CA19-9 levels were significantly associated with distant metastasis, median VAF in baseline ctDNA > 1% was a significant predictor of distant metastasis for patients with normal baseline CEA (HR, 16.130; *p* = 0.001) or CA19-9 levels (HR, 5.379; *p* = 0.005), respectively [48].

Tie et al. reported on the ctDNA dynamics of 159 patients with LARC undergoing neoadjuvant TNT followed by surgery [49]. In this study, the mutation with the highest VAF (relative to normal control DNA) was selected for ctDNA analysis. Baseline ctDNA was detected in 77% of patients, but was only detected in 8.3% and 12% of post-CRT and post-surgery, respectively. Although ctDNA status at baseline and post-CRT was not associated with any clinicopathological factors, detection of ctDNA post-surgery correlated with poorer histopathological staging. Detectable ctDNA post-CRT (HR 6.6; *p* < 0.001) or post-surgery (HR 13.0; *p* < 0.001) were both significant predictors of early recurrence. Indeed, detection of ctDNA post-surgery was a strong predictor of RFS for patients regardless of whether they received adjuvant chemotherapy. Postoperative ctDNA status remained an independent predictor of recurrence-free survival after adjusting for known clinicopathological risk factors (HR 6.0; *p* < 0.001) [49].

In a study of 29 patients with newly diagnosed LARC that received neoadjuvant CRT prior to surgery, McDuff et al. profiled ctDNA status from blood collected at baseline, pre- and post-surgery [56]. NGS was used to identify mutations in the primary tumor, and mutation-specific ddPCR was used to assess the mutation fraction in ctDNA. Although there was no significant correlation between baseline ctDNA positivity and pCR, the proportion of patients that were margin-negative and node-negative after resection was significantly higher among those with undetectable ctDNA pre-surgery compared to ctDNA positive patients (88% vs. 44%; *p* = 0.028). Whether ctDNA status pre-surgery also purported reduced risk of recurrence was not evaluated; however, detectable postoperative ctDNA was associated with poorer RFS (HR 11.56; *p* = 0.007)^56^.

A correlative study stemming from the GEMCAD 1402 trial examined the clinical impact of ctDNA in patients with LARC after receiving trimodal TNT consisting of induction chemotherapy (mFOLFOX6) with or without aflibercept followed by CRT [57]. Without a priori knowledge of tumor mutational status, the presence of somatic alterations and an epigenomic cancer signature was used to determine ctDNA status in blood samples collected from patients at baseline and after completion of TNT. Using a proprietary bioinformatics caller to exclude common sources of interference, such as clonal hematopoiesis of indeterminate potential (CHIP), resultant DNA status was reported as either “detected” or “not detected.” No association was found between ctDNA detected at baseline or pre-surgery (i.e., collected within 48 h prior to surgery), and neither was predictive of treatment response. Compared with patients with negative ctDNA, however, detection of ctDNA pre-surgery was associated with an increased risk of recurrence (HR 4.029; 95% CI: 1.004–16.16; *p* = 0.033) and dramatically reduced survival (HR, 23; 95% CI: 2.4–212; *p* < 0.0001). Indeed, patients with ctDNA detected pre-surgery had 18% lower RFS at 3 years compared to those with ctDNA not detected (66% vs. 84%). At pre-surgery, patients with ctDNA detected and CEA elevated, or those with CEA elevated, had worsened RFS outcomes compared to patients that were negative for both biomarkers (*p* = 0.044). Moreover, pre-surgery ctDNA was detectable in 75% of patients who developed liver metastases during follow-up compared with 9.8% who never developed liver metastases.

Murahashi et al. profiled ctDNA levels in patients with LARC to predict response to pre-operative therapy and post-operative recurrence [58]. Serially collected blood from 85 LARC patients was assessed using amplicon-based deep sequencing of a cfDNA panel covering 14 genes with over 240 hotspots. ctDNA was detected in 57.6% and 22.3% of samples at baseline and pre-surgically, respectively, with nearly two-thirds of the targeted mutations detected at baseline were not observed in pre-surgical samples regardless of response. A ≥ 80% change in ctDNA status (from baseline) was an independent predictor of complete response to neoadjuvant therapy. The positive predictive value for detecting this ≥ 80% change in ctDNA was 33.3% but was increased to 54.5% when combined with endoscopic findings. Examination of post-operative ctDNA and CEA levels showed that each was independent predictors for recurrence after surgery, but a combination of the two biomarkers had a cumulative effect on the RFS. This suggests that a hybrid approach to clinical management after completing NAT or surgery may be warranted [58].

Wang et al. explored the utility of combining ctDNA with MRI as a predictor of pCR pre-surgery and potential for risk stratifying patients with LARC undergoing neoadjuvant CRT and surgery [59]. A target panel of 422 cancer-related genes was evaluated in blood samples collected at baseline, during CRT, and post-surgery from 119 LARC patients. Somatic mutations were present in 84% of patients at baseline, but this was not associated with treatment response or survival. Detection of the mutation with the highest VAF was used as a measure of ctDNA clearance and showed that the proportion patients failing to achieve pCR was significantly lower if ctDNA was detected while receiving neoadjuvant therapy. Likewise, worsening TRG correlated with an incrementally decreasing rate of ctDNA clearance, as well as increased rate of acquired mutations. Extending these findings to include a MRI-based assessment of TRG (mTRG) showed that the ability to predict pCR/non-pCR status was significantly improved by combining with ctDNA monitoring (AUC 0.886, 95CI% 0.810–0.962) compared to using ctDNA (AUC 0.818, 95% CI 0.725–0.912) or mTRG (AUC 0.729, 95% CI 0.641–0.816) alone. The ctDNA status in this model was based on the presence of 5 features: TP53 mutation, mutations in genes associated with homologous recombination repair, mutations in genes associated with histone methyl transferase, as well as both the rate of ctDNA clearance and the rate of acquired mutation during neoadjuvant CRT. Although attaining pCR was a significant RFS predictor, the prognostic value of this ctDNA signature combined with mTRG monitoring for predicting recurrence was not reported. That said, the presence of driver mutations (e.g., TP53, KRAS) in ctDNA detected pre-and post-surgery was significant predictors of recurrence.

Khakoo et al. also examined the combination of ctDNA and mTRG monitoring in a cohort of 47 LARC patients during neoadjuvant CRT [60]. Using ddPCR to examine a targeted panel of somatic mutations (KRAS, NRAS, BRAF, PIK3CA, TP53, and APC) ctDNA levels decreased from 74% at baseline to 21% and 13% post-CRT and post-surgery, respectively. Significantly shorter metastases-free survival was associated with ctDNA persistently detected throughout CRT (HR 11.5, 95% CI, 3.3–40.4) as well as post-CRT (HR 7.1, 95% CI 2.4–21.5). Moreover, ctDNA at baseline was detected in patients with clinically meaningful CEA levels (i.e., ≥ 5 μg/ml) as well as large portion of patients with low CEA levels (i.e., < 5 μg.ml). Using mTRG 1–2 and mTRG 3–5 to dichotomize good and poor responders, respectively, patients with detectable ctDNA post-CRT were more likely to have worsened outcomes. Comparisons of mTRG response with ctDNA status at other timepoints did not show any correlations. Among 23 patients that proceeded to surgery, 20 patients had undetectable ctDNA post-surgery, with DFS being significantly shorter among the 3 patients with detectable ctDNA post-surgery (HR 39.9; 95% CI, 4.0–399.5). Notably, among 15 patients that were non-operatively managed following neoadjuvant CRT, 10 patients experienced localized recurrence, with significantly shorter RFS observed in among those patients with ctDNA detected post-CRT (HR 5.8, 95% CI, 0.9–35.3) [60].

In an attempt to improve the sensitivity of ctDNA monitoring, Boniface et al. examined the feasibility of applying dual-indexed, degenerate adaptor-sequencing (DIDA-Seq) as a novel technology [61]. DIDA-Seq combines unique molecular indexing (UMI)–based error correction with custom hybridization capture at many genomic loci of somatic variants previously identified by whole-exome sequencing of the patient’s tumor tissue, which allows the detection of ctDNA in the blood with very high accuracy (one error in 10 k–50 k observations) and sensitivity (0.005–0.02% minimum variant allele frequency). The pilot study only examined 2 patients with LARC and another 3 patients with esophageal adenocarcinoma undergoing neoadjuvant CRT. For the 2 LARC patients, WES of each patient’s tumor revealed 81 and 106 non-synonymous single nucleotide variations (SNVs), respectively. This subsequently informed DIDA-Seq of 28 and 35 loci in patients 1 and 2, respectively, that was used for ctDNA monitoring. Patient 1 presented with distal rectal cancer (cT3N1M0) and received TNT comprising eight cycles of FOLFOX chemotherapy and CRT that was followed by non-operative management for cCR based on MRI and endoscopy. Using DIDA-Seq of 28 loci to monitor ctDNA showed that ctDNA levels during TNT decreased fivefold, but detectable levels persisted throughout the ~ 6 months of non-operative management following TNT. Disease recurrence was clinically detected 11 months post-TNT, and the patient underwent salvage TME. Patient 2 had mid-rectal adenocarcinoma (cT2N1M0) and underwent TNT (8 cycles FOLFOX and CRT) followed by non-operative management. Per the DIDA-Seq of 35 loci, ctDNA levels at baseline and during TNT were not significantly above background controls; however, mutant reads were continuously present. Elevated ctDNA levels were observed 8 months post-TNT, and 1 month later, the patient showed clinical evidence of local recurrence. ctDNA levels spiked at the time of salvage TME, but again dropped below the limits of detection. This low levels of ctDNA persisted despite the presence of oligometastatic progression in the lungs observed 2 months later. Although in its infancy, the DIDA-Seq offers highly sensitive means for monitoring patient- and tumor-specific ctDNA during and after TNT [61].

### ctDNA Monitoring Beyond Mutations

Identifying tumor-specific mutations remains a key challenge in the optimization of a robust ctDNA monitoring platform for LARC patients. Several studies have sought to overcome this hurdle by examining non-genetic characteristics that may be intrinsic to monitoring tumor response during TNT [46, 47, 62].

Appelt et al. examined the hypermethylation status of the neuropeptide (NPY) gene in serum samples from 146 patients with LARC undergoing TNT [63]. Of these, only 30 patients had detectable methylated NPY ctDNA, but compared to those with no detectable signal, was associated with significantly worsened 5-year OS (HR 2.08 95% CI 1.23–1.51) and disease-free metastases (HR 2.20 95% CI 1.19–4.07). Albeit preliminary, the findings highlight the potential of examining methylation status in cfDNA as a prognostic marker in the neoadjuvant setting.

Liu et al. examined the utility of three different strategies for profiling cfDNA in 60 patients with LARC patients neoadjuvant therapy (NAT) [64]. The first was a personalized assay that employed Mutation Capsule technology to profile up to 22 tumor-informed somatic mutations [65]. In this approach, the estimated ctDNA fraction was based on the allelic fraction and sequencing depth of somatic mutations in tumor tissues and paired plasma samples. The second was a universal panel of 15 genes frequently mutated in colorectal cancer, and the third approach examined the low depth sequencing for copy number alterations (CNAs). Relative comparisons of each approach showed that the accuracy for recurrence prediction was higher when using a personalized assay (sensitivity = 76.47%, specificity = 97.67%) compared to the universal panel (sensitivity = 66.67%, specificity = 78.38%) or baseline-specific (i.e., pre-NAT) CNA (sensitivity = 66.67%, specificity = 100%). Notably, the addition of baseline-specific CNA to either the personalized assay or universal panel significantly improved the accuracy for recurrence prediction. For the personalized 22-gene assay, clearance of ctDNA during NAT was significantly associated with response, whereby the majority (18/19 [94.74%]) of patients not achieving a cCR had failed to clear ctDNA (defined as: $\frac{\mathrm{ctDNA}\;{\mathrm{fraction}}_{\mathrm{post}-\mathrm{NAT}}}{\mathrm{ctDNA}\;{\mathrm{fraction}}_{\;\mathrm{baseline}}}<2\%$). Overall, compared to the ctDNA-negative fraction of patients, those showing ctDNA positivity following NAT had significantly higher risks of recurrence (HR = 27.38; 95% CI, 8.61–87.06, log-rank *p* < 0.0001), distant metastasis-free survival (HR = 17.37; 95% CI, 4.62–65.28; log-rank *p* < 0.0001), and local relapse-free survival (RFS, HR = 20.59; 95% CI, 2.26–187.40; log-rank *p* = 0.00016). This ability to predict risk of RFS was notably improved if the personalized 22-gene assay was combined with baseline-specific measure of CNA (HR = 35.89; 95% CI, 9.93–129.80; log-rank *p* < 0.0001). Moreover, ctDNA positivity after completing TNT was a stronger independent predictor of recurrence post-TNT than either CEA (HR = 1.21; 95% CI, 0.34–4.25; log-rank *p* = 0.77) or CA-19–9 (HR = 1.06; 95% CI, 0.14–8.11; log-rank *p* = 0.96). Notably, for the 17 patients experiencing recurrence, there was median ~ 10 month (range, 0.1–33.2 months) lag time between ascertaining ctDNA positivity post-NAT and detecting radiographic evidence of recurrence. In the context of detecting minimal residual disease (MRD) after completing NAT, a lower tumor fraction and corresponding low cfDNA abundance can limit sensitivity of detecting point mutations [62, 66]. CNA makes up a large proportion of the cfDNA, and integration with mutational profiling can optimize the sensitivity for estimating the tumor fraction [62, 66].

Using a previously reported cohort of 119 LARC patients, Wang et al. separately reported on the value of cfDNA fragmentomics to predict pCR [67]. The study examined four discrete ranges of cfDNA fragments (i.e., 1–64, 65–99, 100–220, 221–400 bp) along with differing lengths of the 5′-end motif (i.e., 4-mer, 5-mer, 6-mer). The latter of these DNA fragment characteristics stems from preservation of the 5′ protruding ends in the Watson and Crick strands of plasma DNA [68]. Using elastic net logistic regression modeling, detection of 6-mer 5′-end motif was consistently better at predicting pCR than either 4-mer or 5-mer 5′-end motifs and displayed superior performance to predicting pCR than fragment profiling. Combined with mTRG, 5′-end motif profiling was a significant predictor of non-pCR (AUC 0.92, 95% 0.91–0.93) and yielded higher cross-validation AUC than 5′-end motif alone (AUC 0.90, 95% CI 0.89–0.91) or in combination with ctDNA detection (AUC 0.91, 95% CI 0.90–0.92). Moreover, in patients without detectable ctDNA, profiling of 5′-end motif alone or in combination with mrTRG was a strong predictor of non-pCR. Without altering sensitivity, the specificity of predicting response using 5′-end motif was increased from 83 to 87.5% when combined with mTRG. These findings suggest that after completing neoadjuvant treatment, assessment of 5′-end motifs status may provide a useful tool for predicting response in the absence of detectable ctDNA.

## Discussion

Efforts to optimize the clinical management of LARC have continued to evolve with improvements in neoadjuvant strategies (i.e., TNT), as well as a growing clinical impetus for non-operative management of patients that achieve cCR [5, 6, 69, 70]. This shift in the clinical paradigm, however, has underscored the urgent need for robust biomarkers to monitor clinical response throughout TNT and predict the likelihood of disease recurrence. In this regard, liquid biopsies and the detection of cfDNA/ctDNA from the plasma of cancer patients are an emerging technology with the potential to provide a non-invasive approach to monitor disease response and improve the identification of patients that would best benefit from non-operative management. Even so, substantial work is still needed before cfDNA/ctDNA monitoring can be widely adopted in the clinical setting, and the studies reviewed herein highlight several areas of opportunity for improving the effectiveness and utility of cfDNA/ctDNA for managing patients with LARC.

Are changes in ctDNA levels predictive of cCR and non-operative management following TNT? In general, levels of ctDNA appear dynamic during the course of TNT, typically starting with high pre-treatment/baseline levels that experience fold-change decreases during TNT [48, 56, 58, 59]. While higher levels of ctDNA at baseline are not reliably prognostic of response, persistent levels of cfDNA/ctDNA detected during and after NAT or prior to surgery correlate with shortened time to disease recurrence, and has given rise to the concept of utilizing ctDNA as a biomarker of minimal metastatic disease (MMD) [36, 37, 48, 56, 57, 60, 64]. Correlations between detectable ctDNA post-NAT and mTRG further point to the practicability of combining ctDNA with image-based monitoring strategies [56, 59, 60, 67].

The majority of the above studies reported on neoadjuvant CRT, with few examining cfDNA/ctDNA dynamics when systemic chemotherapy is incorporated into the treatment regimen. Moreover, most patients in these studies underwent surgical resection, leaving the question of non-operative management unclear. Whether ctDNA can directly inform non-operative management will require additional examination of ctDNA dynamics throughout TNT and is the focus of several on-going studies (Table 1).

**Table 1:** Summary of ongoing studies evaluating the utility of ctDNA in patients with LARC

**NCT number**	**Title**	**Study type/intervention**	**Disease**	***N***	**Status**	**Outcome measures**	**Sponsor/collaborators**
**NCT05601505**	Circulating Tumor DNA-guided Neoadjuvant Treatment Strategy for Locally Advanced Rectal Cancer (CINTS-R)	Interventional ctDNA-based randomization: VAF < 0.4% receive neoadjuvant CRT vs VAF > 0.4% receive TNT	LARC	465	Recruiting	**Primary outcome:** Disease-related treatment failure (DrTF) **Secondary outcomes:** Time to recurrence, OS	Peking Union Medical College Hospital
**NCT05081024**	Establishing a ctDNA Biomarker to Improve Organ Preserving Strategies in Patients With Rectal Cancer (ctTRAC)	Observational n/a	LARC	50	Recruiting	**Primary outcome:** cCR **Secondary outcomes:** Positive ctDNA, rate of transabdominal surgery, rate of pCR, rate of W&W, DFS, OS	OHSU Knight Cancer Institute Natera, Inc
**NCT04842006**	Systemic Neoadjuvant and Adjuvant Control by Precision Medicine in Rectal Cancer (SYNCOPE)	Interventional Randomization: TNT short-RT + capecitabine/oxaliplatin vs long-RT + capecitabine	Colorectal cancer	93	Recruiting	**Primary outcome:** RFS **Secondary outcomes:** Postoperative ctDNA, CRC-specific survival, OS, number of surgically resected patients resected patients, R0-resection rate, local recurrence rate, pCR rate, cCR rate, total uptake of chemotherapy, AEs	Helsinki University Central Hospital
**NCT03916510**	Chemoradiation With Enadenotucirev as a Radiosensitiser in Locally Advanced Rectal Cancer (CEDAR)	Interventional Enadenotucirev + capecitabine + RT	LARC	30	Recruiting	**Primary outcomes:** DLTs + mTRG **Secondary outcomes:** pCR, neoadjuvant Rectal (NAR) score Recurrence rates, ctDNA levels	University of Oxford|PsiOxus Therapeutics Ltd|Cancer Research UK
**NCT03714490**	MRI Simulation-guided Boost in Short-course Preoperative Radiotherapy for Unresectable Rectal Cancer (SUNRISE)	Interventional Randomization: Neoadjuvant short-RT capecitabine + oxaliplatin –TME vs CRT TME	Rectal cancer	200	Recruiting	**Primary outcome** R0 rate **Secondary outcome** Local RFS, DFS. OS. pCR, AEs, QOL **Exploratory outcome** ctDNA levels (509-gene panel)	Cancer Institute and Hospital, Chinese Academy of Medical Sciences
**NCT03615170**	Application of Circulating Tumor DNA Test in the Diagnosis and Treatment of Patients With Advanced Rectal Cancer	Observational n/a	Rectal cancer	200	Recruiting	**Primary outcome** DFS	Zhejiang Cancer Hospital
**NCT03565029**	Total Neoadjuvant Treatment Without Surgery For Locally Advanced Rectal Cancer (NO-CUT)	Interventional Neoadjuvant oxaliplatin-based chemotherapy CRT	LARC	180	Recruiting	**Primary outcome:** Distant relapse-free survival (DRFS) **Secondary outcomes:** cCR rate Local recurrence rate, organ preservation rate Colostomy-free survival, OS, QOL	Niguarda Hospital
**NCT03484221**	Totally Neoadjuvant FOLFOXIRI + Short-course Radiation + XELOX in Patients With Locally Advanced Rectal Cancer	Interventional Neoadjuvant FOLFOXIRI short-course RT + capecitabine	LARC	30	Recruiting	**Primary outcome:** Ratio of tumor downstaging to stage 0 and stage I **Secondary outcomes:** TRG rate, DFS, OS, TEAEs, ctDNA levels, SUVmax changes, QOL	China Medical University, China
**NCT02992886**	Preoperative Chemoradiotherapy With Raltitrexed for Intermediate or Locally Advanced Rectal Cancer in the Fit Elderly	Interventional CRT + Raltitrexed TME	LARC	68	Completed	**Primary outcome:** 2-year DFS **Secondary outcomes:** 5-year OS, 5-year cancer-specific survival, pCR rate, AEs, QOL **Exploratory outcomes** ctDNA levels	Cancer Institute and Hospital, Chinese Academy of Medical Sciences
**ACTRN126150-00,381,583**	A study to evaluate the use of circulating tumor DNA to guide adjuvant chemotherapy on recurrence-free survival in patients with stage II Colon or rectal cancer (DYNAMIC)	Interventional Randomization (2:1): Arm A—ctDNA +, receive adjuvant chemo vs. ctDNA-, no chemotherapy vs Arm B—ctDNA agnostic, physician’s choice standard of care	Colorectal cancer	450	Active, not recruiting	**Primary outcome:** RFS **Secondary outcomes:** OS, change in serial ctDNA during treatment with disease recurrence	Royal Melbourne Hospital

Optimizing cfDNA/ctDNA detection remains a crucial hurdle as there is a repeated trend of not detecting ctDNA in ~ 15–25% of patients [48, 49, 56, 57, 59]. This is compounded by the possibility that mutations detected at baseline may not be present post-TNT, and cautions against using minimally selective targeted mutational panels [48]. Overcoming the low tumor burden that is intrinsic to rectal cancer may require employing technologies with higher ctDNA sensitivity of mutational detection (e.g., DIDA-seq) that can be combined with non-genetic strategies (e.g., fragmentomics) [61, 67]. As it stands currently, there are no published ctDNA risk-adapted studies in managing patients undergoing total neoadjuvant therapy in hopes of pursuing organ preservation. One useful trial design would use a ctDNA biomarker-driven risk-adaptive approach whereby patients with cCR that have residual ctDNA are randomized to non-operative management versus TME.

Can cfDNA/ctDNA determine the need for adjuvant therapy following TNT and/or TME? In addition to the lack of ctDNA clearance post-NAT, several studies reported that post-surgical detection of ctDNA in patients with LARC is also associated with a shorter time to recurrence [48, 49, 58, 60]. The utility of post-TNT or post-TME ctDNA in guiding adjuvant therapy may be further enhanced by risk stratification based on the detection of specific driver mutations (e.g., TP53, KRAS) associated with higher rates of recurrence, as well as combined with clinicopathological features (e.g., perineural invasion, tumor deposits, vascular invasion, and lymph node metastasis) that altogether portend worsened outcomes [49, 59]. The basis for ctDNA as a biomarker for predicting adjuvant treatment intensification in colorectal cancer is ongoing [71]. Results from the DYNAMIC trial may help to address this question, in which ctDNA status after completion of neoadjuvant CRT and surgery is used to guide adjuvant chemotherapy (Table 1) [72]. Similarly, the BESPOKE trial (NCT04264702) is using the Signatera assay to monitor patients in the adjuvant setting and determine the rate of recurrence of patients with colorectal cancer while asymptomatic. The significance here would be to help detect patients who would best benefit from intervention of their oligometastatic disease early.

Is there an optimal cfDNA/ctDNA signature to inform on the type and intensity of chemotherapy or radiotherapy components comprising TNT? A penultimate achievement in precision oncology would be for cfDNA/ctDNA to serve as a non-invasive approach for repeated, serial measures capable of identifying targetable mutations or altered signaling pathways, which in turn would inform specific types of treatments. The precedence for such an approach has been reported in other cancer types, but remains to be determined in rectal cancer [32]. The SYNCOPE trial (Table 1) in Finland will randomize LARC patients to either TNT defined as short course RT plus capecitabine/oxaliplatin or long course CRT. The aim is that ctDNA levels will reveal patients who can undergo treatment de-escalation without compromising oncologic outcomes.

Cercek et al. recently reported on the clinical outcomes of 12 patients with high-levels of microsatellite instability (MSI-H) LARC that received the PD-1 immune checkpoint blocker, dostarlimab, in the neoadjuvant setting [73]. Remarkably, single agent treatment with dostarlimab achieved a cCR rate of 100% (95% CI, 74–100%) after at least 6 months of follow-up. At last follow-up, none of the 12 patients had undergone CRT or surgery; no cases of progression or recurrence were reported. While validation of these findings is still needed, this treatment strategy represents a significant boon to managing MSI-H LARC patients. Even so, the treatment only benefits a small portion of patients as the majority of rectal cancers are MSI stable (MSI-S, ~85%) [74]. In this regard, Zhou et al. observed an improved response to neoadjuvant CRT in LARC patients with detectable ctDNA comprised of the polymerase epsilon delta 1 (POLD1) mutation, and noted that TMB was significantly higher in patients harboring POLD1 mutations [48]. POLD1 is a protein critical for proofreading and fidelity in DNA replication, and POLD1 mutations are positive predictors for the survival benefit from immune checkpoint blockers, as well as exceptional chemotherapy response in a metastatic colorectal cancer patient [48, 75, 76]. Separately, Ji et al. reported that high bTMB levels at baseline were associated with increased immune activity, as measured by elevation in serum cytokines (IFNγ, IFNα2, IL-1β, IL-2, and MIP-1β), and low bTMB levels at baseline corresponded with an increased risk of recurrence [55]. Altogether, these findings provide the basis by which ctDNA monitoring may aid in identifying additional rectal cancer patients (beyond MSI-H) that could benefit from similar immunotherapy strategies.

## Conclusions

It is important to note that most of the studies reported have been exploratory with small numbers of patients that limit statistical inferences. This is further compounded by the wide variation of cfDNA/ctDNA detection strategies that limit generalization of the reported findings. Even so, other liquid biopsy technologies being developed (e.g., extracellular vesicles, cfRNA, circulating tumor cells) are still emerging, and cfDNA/ctDNA is currently the most advanced biomarker available to guide TNT for rectal cancer [77–79]. As technologies improve, and more rigorous trials are conducted, it is likely that non-operative management may be more comfortably employed if a validated cfDNA/ctDNA biomarker is readily available to optimize the identification of responding patients that would best benefit under the watchful waiting paradigm. Likewise, reliable detection of cfDNA/ctDNA for non-responders would be a welcomed biomarker for identifying patients that may benefit from treatment intensification.
